# Dual Protective Effects of Postbiotics and *Cichorium intybus* L. Mixture in the Sarcopenic and Inflammatory Models

**DOI:** 10.3390/biomedicines13092046

**Published:** 2025-08-22

**Authors:** Harang Park, Jinsu Choi, Eunwoo Jeong, Hye-Yeong Song, Juyeong Moon, Min-ah Kim, Chunghyeon Lee, Junsoo Park, Jong Kwang Hong, Tack-Joong Kim

**Affiliations:** 1 Division of Biological Science and Technology, Yonsei University, Wonju 26493, Republic of Korea; phraa@naver.com (H.P.); wlstnbnm@naver.com (J.C.); jew0108@naver.com (E.J.); shy9987@naver.com (H.-Y.S.); mjy@yonsei.ac.kr (J.M.); mina1218@yonsei.ac.kr (M.-a.K.); ichmaru@yonsei.ac.kr (C.L.); junsoo@yonsei.ac.kr (J.P.); jongkwang.hong@yonsei.ac.kr (J.K.H.); 2 Research & Development Center, Doctor TJ Co., Ltd., Wonju 26494, Republic of Korea

**Keywords:** aging, beLP1, *Cichorium intybus* L. root extract, inulin, inflammation, DuoX, rat animal model, sarcopenia

## Abstract

**Background/Objectives**: Recently, concerns about age-related conditions, such as sarcopenia and chronic inflammation, have increased owing to the global acceleration of population aging. Notably, these conditions are interrelated and further exacerbate functional decline in older adults. Therefore, this study aimed to evaluate the efficacy of a novel bioactive compound, DuoX (a mixture of the postbiotic beLP1 and *Cichorium intybus* L.), in alleviating muscle wasting and chronic inflammation. Specifically, the mixture consisted of inulin-rich *C. intybus* L. root extract, known for its anti-inflammatory effects, and beLP1, a postbiotic previously shown to exert anti-sarcopenic effects. **Methods**: To assess the multifunctional effects of the DuoX, dexamethasone-induced sarcopenia models (C2C12 myotubes and an in vivo rat model) and a lipopolysaccharide-stimulated RAW 264.7 macrophage inflammation model were established. **Results**: Pretreatment with DuoX prevented the dexamethasone-induced reduction in myotube diameter and effectively inhibited muscle degradation by downregulating the expression of atrogin-1 caused by dexamethasone treatment. In rats with DEX-induced sarcopenia, DuoX prevented muscle weight loss, grip strength reduction, and the upregulation of atrogin-1 expression in vivo. In lipopolysaccharide-stimulated RAW 264.7 macrophages, DuoX significantly reduced nitric oxide production and cyclooxygenase-2 protein expression and suppressed p38 and ERK phosphorylation in the MAPK signaling pathway, thereby alleviating inflammatory responses. **Conclusions**: DuoX holds promise as a dual-functional candidate with both anti-sarcopenic and anti-inflammatory properties. Further preclinical and clinical studies are required to validate its therapeutic efficacy and safety in humans, which may contribute to the development of preventive strategies for healthy aging.

## 1. Introduction

Over the past few decades, the increase in average life expectancy has led in older adults to a rise in various age-related diseases, including sarcopenia, as well as a decline in physical function [1,2]. Sarcopenia is a progressive systemic skeletal muscle disorder characterized by a gradual loss of muscle mass and strength, which is closely associated with impaired physical function, increased hospitalization rates, and elevated mortality in older populations [3,4,5,6].

Aging induces the accumulation of reactive oxygen species and mitochondrial damage, resulting in oxidative stress and impaired energy metabolism, ultimately causing muscle fiber atrophy and decreased muscle strength [7]. Additionally, aging is accompanied by decreased synthesis and increased degradation of muscle proteins, with the ubiquitin-proteasome system (UPS)-mediated breakdown via atrogin-1 playing a key role [8,9].

Inflammation serves as a defense response; however, chronic low-grade inflammation termed “inflammaging” persists with aging, leading to tissue damage and an increased risk of disease [10,11]. This phenomenon is explained by the antagonistic pleiotropy theory, which suggests that beneficial inflammatory responses in youth become detrimental during aging [10,12]. Inflammatory responses are primarily mediated through the mitogen-activated protein kinase (MAPK) and nuclear factor kappa-light-chain-enhancer of activated B cells (NF-κB) signaling pathways [13,14]. Notably, elevated levels of pro-inflammatory cytokines have been linked to an increased risk of sarcopenia [15,16].

Dexamethasone (DEX), a synthetic glucocorticoid, is widely used in preclinical models to induce sarcopenia due to its ability to suppress muscle protein synthesis and promote proteolysis [17,18,19]. Lipopolysaccharide (LPS) is a representative pro-inflammatory agent that activates NF-κB and MAPK pathways via the Toll-like receptor 4, leading to the production of various inflammatory cytokines and mediators [20,21,22,23,24,25].

Postbiotics are defined as “preparations of inanimate microorganisms and/or their components that confer health benefits to the host.” Owing to their lack of infection risk and exemption from stringent regulatory requirements, postbiotics can be safely derived from a wide variety of microorganisms [26,27,28,29]. Previous studies have investigated the effects of postbiotics on sarcopenia [30,31,32,33,34,35,36]. Especially, the postbiotic strain *Lactobacillus plantarum* beLP1, isolated from kimchi, has been shown in previous studies to exert anti-sarcopenic effects by regulating the expression of MuRF1 and atrogin-1 through the AKT pathway [37].

In addition, plant extracts containing bioactive compounds such as polyphenols and carotenoids are widely regarded as safe alternatives to synthetic agents, owing to their well-established antioxidant and anti-inflammatory properties [27]. Among these, *Cichorium intybus* L. (chicory) root is a natural source rich in inulin, a functional dietary fiber officially recognized for its safety by the U.S. Food and Drug Administration (FDA) in 2003. Inulin exerts significant antioxidant and anti-inflammatory effects within the gut environment. In particular, inulin inhibits NF-κB activation, thereby reducing the expression of the downstream inflammatory mediator gene of cyclooxygenase-2 (COX-2), which consequently decreases the production of COX-2–dependent pro-inflammatory prostaglandins [38].

Here, we aimed to evaluate the dual preventive potential of DuoX, a functional compound combining the postbiotic strain *L. plantarum* isolated from kimchi and chicory root extract. We assessed the preventive effects of DuoX against DEX-induced sarcopenia in C2C12 myotubes and a rodent sarcopenia model, as well as against LPS-induced inflammatory responses in a RAW 264.7 macrophage model, to elucidate the molecular mechanisms underlying the anti-sarcopenic and anti-inflammatory effects of DuoX.

## 2. Materials and Methods

### 2.1. DuoX Formulation

DuoX is a composite bioactive formulation comprising the following components by weight percentage: Heat-killed postbiotic beLP1 (8.32%), inulin derived from *C. intybus* L. root extract (9.97%), Heat-killed postbiotic EF-2001 (3.34%), Heat-killed postbiotic beLP3 (1.64%), resistant maltodextrin (14.94%), α-brown rice powder (9.97%), mixed grain powder (9.97%), crystalline dextrose powder (4.98%), maltodextrin (4.98%), fructooligosaccharide (16.94%), and fermented rice powder (14.95%). The postbiotic beLP1 used in this study was provided by Bereum Co., Ltd. (Wonju, Republic of Korea), and the inulin derived from *C. intybus* L. root extract was supplied by Beneo-Orafti S.A. (Tienen, Belgium). All components were thoroughly blended to obtain a homogeneous powder mixture, which was subsequently utilized in all experimental assays.

### 2.2. Establishment of the Cellular Sarcopenia and Inflammation Models

C2C12 mouse myoblasts were obtained from the American Type Culture Collection (ATCC, Manassas, VA, USA) and cultured in high-glucose Dulbecco’s modified Eagle’s medium (DMEM; Sigma-Aldrich, St. Louis, MO, USA) supplemented with 10% (*v*/*v*) fetal bovine serum (FBS; Access Biologicals LLC, Vista, CA, USA) and 1% (*v*/*v*) penicillin-streptomycin (Sigma-Aldrich). Cells were maintained at 37 °C in a humidified atmosphere containing 5% CO_2_. For differentiation into myotubes, cells at approximately 90% confluency were transferred to a differentiation medium containing 2% horse serum (Thermo Fisher Scientific Inc., Waltham, MA, USA) and 1% (*v*/*v*) penicillin-streptomycin (Sigma-Aldrich) for 6 days, and the medium was replaced with fresh medium every 2 days. Stock solutions of DuoX (10 mg/ml) and DEX (10mM) were prepared by diluting DuoX in distilled water (DW) and DEX in phosphate-buffered saline (PBS) prior to further dilution in the differentiation or serum-free media used for the C2C12 myotube experiments. DuoX diluted with the differentiation medium was added to the cells after 4 days of differentiation at 100, 200, and 500 μg/mL. After 48 h of DuoX treatment, 100 μM DEX (Sigma-Aldrich) diluted in serum-free medium was applied to the C2C12 myotubes to establish a C2C12 myotube atrophy model.

RAW 264.7 cells (ATCC) were cultured in low-glucose DMEM (Welgene, Inc., Gyeongsan, Republic of Korea) supplemented with 10% (*v*/*v*) FBS (Access Biologicals LLC) and 1% (*v*/*v*) penicillin-streptomycin (Sigma-Aldrich). Cells were maintained at 37 °C in a humidified atmosphere containing 5% CO_2_. In this study, stock solutions of DuoX and LPS (Sigma-Aldrich) were prepared separately, with DuoX dissolved in DW and LPS dissolved in PBS. DuoX was further diluted in serum-free medium and used to pretreat RAW 264.7 cells at varying concentrations (0, 125, 250, and 500 μg/mL) for 30 min, after which the cells were stimulated with 1 μg/mL LPS for 24 h to establish a RAW 264.7 macrophage inflammation model.

### 2.3. Measurements of Myotube Diameters

For morphological analysis, C2C12 cells were stained using May–Grünwald and Giemsa solutions (both Sigma-Aldrich). Before staining, cells were gently washed with cold PBS, fixed in 100% methanol at −20 °C for 5 min, washed with PBS, and then dried at room temperature (20–22 °C). Thereafter, the myotubes were incubated with May–Grünwald solution at 21 ± 3 °C for 5 min, rinsed with PBS, and then stained with Giemsa solution for 15 min under the same conditions. Stained cells were visualized under an optical microscope (Nikon, Tokyo, Japan) at ×200 magnification. Myotube diameters were quantified using the ImageJ software (64-bit Java 1.8.0.170; National Institutes of Health, Bethesda, MD, USA) by measuring 100 myotubes randomly selected from at least ten different viewing fields. 

### 2.4. Animal Experiments

To confirm the in vitro findings, we designed an in vivo study using a rat model of DEX-induced sarcopenia to evaluate the DuoX effects. Six-week-old male Sprague-Dawley rats (average weight: 180 g) were obtained from DBL Korea, Inc. (Eumseong, Republic of Korea). All animals were housed in a pathogen-free environment under controlled conditions (temperature, 23 °C; humidity, 50%; photoperiod, 12 h/12 h light/dark cycle) and provided with standard rodent chow (RodFeed; DBL Inc., Eumseong, Republic of Korea) and sterilized water ad libitum. After a 9-day acclimatization period, the rats were randomly divided into four groups (n = 6 per group) using a simple randomization method to ensure unbiased group allocation, and each rat was individually numbered, and group assignments were determined by a random number generator. The groups were as follows: a normal group (NOR; received oral DW and intraperitoneal injection of saline, without sarcopenia induction); a control group (CON; received oral DW for 19 days and intraperitoneal injection of DEX diluted in saline at 600 μg/kg from day 15 to day 19); low-dose DuoX group (L-DuoX; received oral DuoX diluted in DW at 5000 μg/kg for 19 days along with DEX diluted in saline during the final 5 days); and high-dose DuoX group (H-DuoX; received oral DuoX diluted in DW at 10,000 μg/kg for 19 days, along with DEX diluted in saline during the final 5 days). On the final day, animals were euthanized via CO_2_ inhalation, and the gastrocnemius (GA), tibialis anterior (TA), soleus (SOL), and plantaris (PLA) muscles were removed, weighed, and stored at −80 °C for further analyses. All procedures involving animals were reviewed and approved by the Institutional Animal Care and Use Committee of Yonsei University Mirae Campus (Approval No. YWCI-202504-007-02).

### 2.5. Grip Strength Test

To evaluate the muscle function-preserving effects of DuoX, grip strength was measured using a grip strength meter (Jeongdo Bio & Plant Co., Seoul, Republic of Korea). During the measurement, each rat was allowed to grasp the meter, and grip strength was recorded while gently pulling the rat backward by the tail. The grip strength test was conducted one day after the final treatment with DEX and DuoX, and each rat underwent three measurements.

### 2.6. Nitric Oxide (NO) Assay

RAW 264.7 cells were seeded in 24-well plates and pretreated with DuoX diluted in serum-free medium as described above. After 30 min, the cells were stimulated with LPS, and the culture supernatants were collected and mixed with an equal volume of Griess reagent. After incubating at room temperature (20–22 °C) for 10 min, absorbance was measured at 540 nm using a FLx800 microplate reader (BioTek Instruments Inc., Winooski, VT, USA). The nitrite concentration was calculated based on a sodium nitrite (NaNO_2_) standard curve (0–50 μM).

### 2.7. Protein Extraction and Western Blotting

For in vitro analysis, differentiated C2C12 myotubes were pretreated with DuoX diluted in differentiation medium for 48 h, followed by treatment with 100 μM DEX diluted in serum-free medium for 24 h to induce muscle atrophy. Thereafter, cells seeded in 6-well plates were lysed using PRO-PREP™ Protein Extraction Solution (iNtRON Biotechnology Inc., Seongnam, Republic of Korea). Similarly, RAW 264.7 macrophages were pretreated with DuoX diluted in serum-free medium for 30 min, followed by stimulation with LPS for 24 h. Thereafter, the cells were lysed with PRO-PREP™ Protein Extraction Solution (iNtRON Biotechnology Inc.) for protein analysis. In the in vivo experiment, GA muscles were excised from each experimental group, flash-frozen in liquid nitrogen, and stored at −80 °C until use. Before protein extraction, frozen tissues were homogenized in PRO-PREP™ buffer (iNtRON Biotechnology Inc.) and centrifuged to remove cellular debris. Thereafter, the resulting supernatants were collected for protein quantification. Protein concentrations were determined using the Bradford assay (Bio-Rad, Hercules, CA, USA). Thereafter, equal amounts of protein were separated via SDS-PAGE and transferred onto membranes. After blocking with a blocking buffer, the membranes were incubated at −4 °C overnight with diluted primary antibodies (1:2500 dilution ratio) targeting atrogin-1 (ab168372, Abcam, Cambridge, UK), phosphorylated extracellular signal-regulated kinase (p-ERK; #4377), ERK (#4695), p-p38 (#9211), p38 (#9212), COX-2 (#4842), and GAPDH (#5174, all Cell Signaling Technology, Danvers, MA, USA). Thereafter, the membranes were washed three times with TBS-T and incubated at 20–22 °C for 1 h with a diluted horseradish peroxidase-conjugated secondary antibody (1:5000; anti-rabbit IgG, #7074, Cell Signaling Technology, Boston, USA). After three washes, chemiluminescent signals were developed using WestGlow™ PICO PLUS substrate (Biomax Inc., Guri, Republic of Korea). Imaging was performed using a ChemiDoc™ Imaging System (Bio-Rad), and band intensities were quantified using the ImageJ software. Protein expression levels were normalized to those of GAPDH, p38, and ERK (internal control).

### 2.8. Statistical Analysis

All data analyses were performed using GraphPad Prism (Version 5.02; GraphPad Software, Boston, MA, USA). Significant differences among groups were determined using one-way analysis of variance, followed by Dunnett’s multiple comparison tests. Statistical significance was set at *p* < 0.05, 0.01, 0.001. All experiments were conducted in triplicate. Data are expressed as mean ± standard error.

## 3. Results

### 3.1. DuoX Increases the Dexamethasone-Induced Reduction in Myotube Diameter in C2C12 Cells

**Figure 1:**
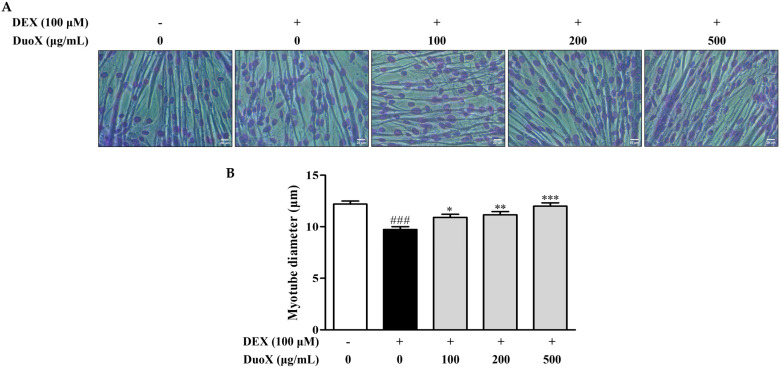
Effects of DuoX on myotube diameter in dexamethasone (DEX)-induced C2C12 cells. (**A**) Representative images of Giemsa-stained myotubes (scale bars: 20 μm). C2C12 cells were cultured in horse serum-containing medium for 6 days to induce differentiation and were pretreated with various concentrations of the DuoX starting on day 5. After differentiation, sarcopenia was induced by treating the cells with DEX for 24 h. (**B**) Measurement of myotube diameters based on Giemsa-stained images. Data are presented as mean ± standard error of mean (n = 100). ### *p* < 0.001 compared with the DEX-untreated control, * *p* < 0.05, ** *p* < 0.01, *** *p* < 0.001 compared with the DEX-treated group.

To evaluate the preventive effects of DuoX on DEX-induced sarcopenia, differentiated C2C12 myotubes were pretreated with or without DuoX and treated with DEX. DEX treatment significantly reduced the mean myotube diameter, indicating muscle wasting. However, pretreatment with DuoX effectively prevented the DEX-induced decrease in myotube diameter, suggesting its potential role in maintaining myotube integrity under catabolic conditions (Figure 1).

### 3.2. DuoX Decreases Atrogin-1 Protein Expression in Dexamethasone-Induced C2C12 Cells

**Figure 2:**
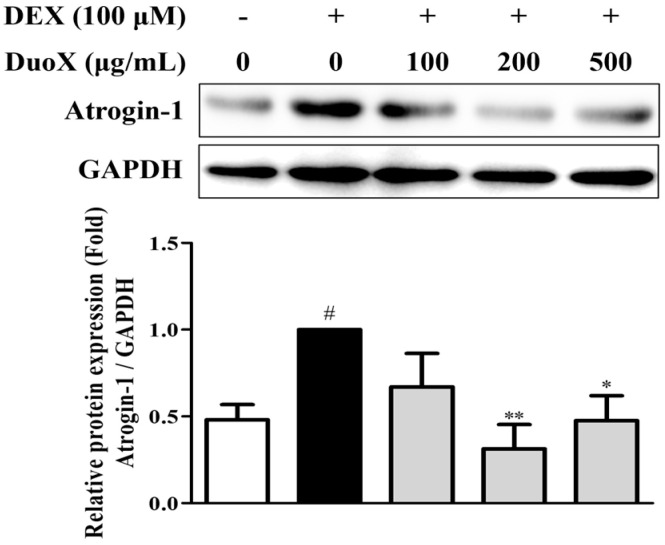
Effects of DuoX on atrogin-1 protein expression in dexamethasone (DEX)-induced C2C12 cells. C2C12 cells were cultured in horse serum-containing medium for 6 days to induce differentiation and were pretreated with various concentrations of DuoX starting on day 5. After differentiation, sarcopenia was induced by treating the cells with DEX for 24 h. Cell lysates were collected and analyzed using western blotting to assess atrogin-1 expression. GAPDH was used as a loading control. Data are presented as mean ± standard error of mean (n = 4). # *p* < 0.05 compared with the DEX-untreated control, * *p* < 0.05, ** *p* < 0.01 compared with the DEX-treated group.

To investigate the effects of DuoX on muscle protein degradation, the expression of atrogin-1, a muscle-specific ubiquitin ligase, in C2C12 myotubes was examined. DEX treatment markedly upregulated atrogin-1 expression, whereas pretreatment with DuoX significantly downregulated its expression. Collectively, these results indicate that DuoX prevents muscle protein degradation by modulating atrogin-1 expression in sarcopenic conditions (Figure 2).

### 3.3. DuoX Prevents Muscle Weight Loss in a Rat Model of Dexamethasone-Induced Sarcopenia

**Figure 3:**
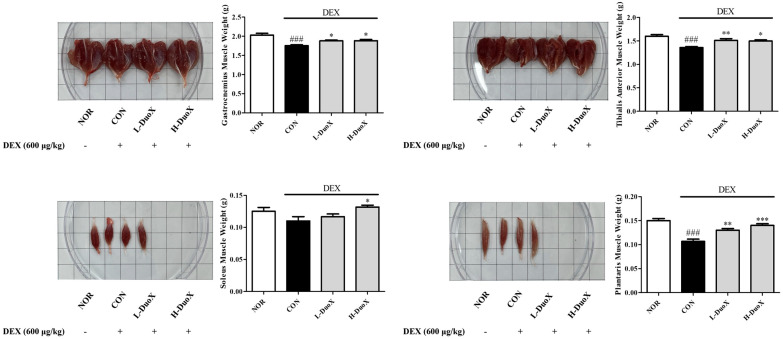
Effects of DuoX on muscle weights in the rats model of dexamethasone (DEX)-induced sarcopenia. Rats were treated with DEX to induce muscle atrophy and were orally administered DuoX at the indicated doses before and during DEX treatment. After the treatment period, the weights of the gastrocnemius, tibialis anterior, soleus, and plantaris muscles were measured. Data are presented as mean ± standard error of mean (n = 6 per group). ### *p* < 0.001 compared with the DEX-untreated control (NOR); * *p* < 0.05, ** *p* < 0.01, *** *p* < 0.001 compared with the DEX-treated group (CON). NOR, normal group; CON, control group; L-DuoX, low-dose DuoX group; H-DuoX, high-dose DuoX group.

To evaluate the protective effect of DuoX against muscle mass loss in vivo, rats were pretreated with or without the mixture and treated with DEX. At the end of the experimental period, the GA, TA, SOL, and PLA muscles were weighed. DEX significantly reduced GA, TA, and PLA muscle weights compared with those in the control group. However, treatment with DuoX prevented DEX-induced decrease in the weights of the muscles, indicating that DuoX may alleviate catabolic stress-induced muscle atrophy in vivo (Figure 3).

### 3.4. DuoX Improves Grip Strength in the Rat Model of Dexamethasone-Induced Sarcopenia

**Figure 4:**
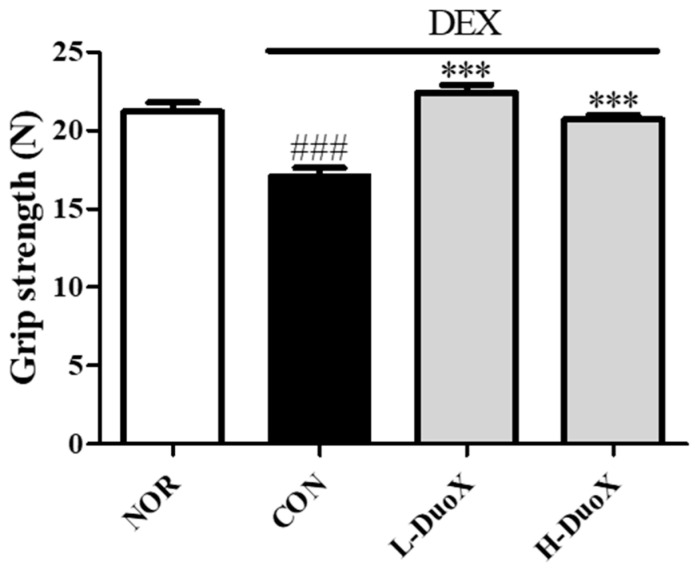
Effects of DuoX on grip strength in the rats model of dexamethasone (DEX)-induced sarcopenia. Rats were treated with DEX to induce sarcopenia and were orally administered DuoX before and during DEX administration at the indicated doses. Grip strength was measured after DEX treatment. Data are presented as mean ± standard error of mean (n = 6 per group). ### *p* < 0.001 compared with the DEX-untreated control (NOR); *** *p* < 0.001 compared with the DEX-treated group (CON). NOR, normal group; CON, control group; L-DuoX, low-dose DuoX group; H-DuoX, high-dose DuoX group.

To assess the functional impact of DuoX on muscle strength, grip strength tests were performed on rats with DEX-induced muscle atrophy. DEX treatment significantly decreased forelimb grip strength compared with that in the control group. However, oral administration of DuoX before and during the DEX treatment period ameliorated the decline in grip strength. Overall, these results suggest that DuoX not only preserves muscle mass but also improves muscle function (Figure 4).

### 3.5. DuoX Decreases Atrogin-1 Protein Expression in the Rat Model of Dexamethasone-Induced Sarcopenia

**Figure 5:**
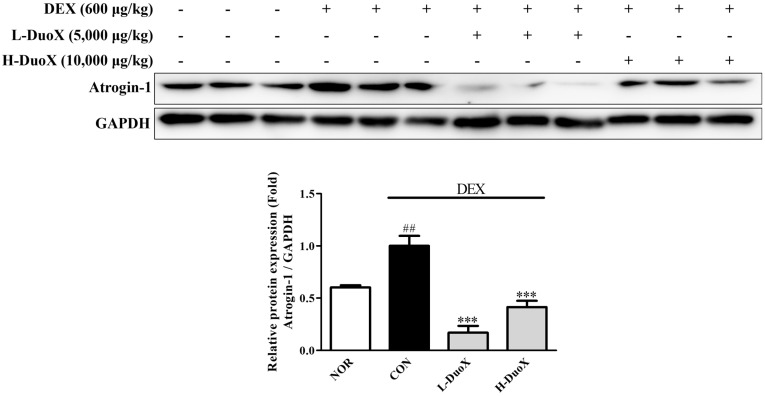
Effects of DuoX on atrogin-1 protein expression in the rats model of dexamethasone (DEX)-induced sarcopenia. Muscle tissues were removed from rats treated with DEX and DuoX. atrogin-1 protein levels were analyzed using western blotting with GAPDH as a loading control. Data are presented as mean ± standard error of mean (n = 3). ## *p* < 0.01 compared with the DEX-untreated control (NOR); *** *p* < 0.001 compared with the DEX-treated group (CON). NOR, normal group; CON, control group; L-DuoX, low-dose DuoX group; H-DuoX, high-dose DuoX group.

To explore the molecular mechanism underlying the anti-sarcopenic effects of DuoX in vivo, the expression of atrogin-1 in skeletal muscle tissues was analyzed. DEX-treated rats showed elevated atrogin-1 levels in their skeletal muscles compared with those in the control group. However, oral administration of DuoX significantly suppressed the DEX-induced increase in atrogin-1 levels in skeletal muscles. Collectively, these results suggest that DuoX prevents muscle protein degradation by downregulating atrogin-1 expression (Figure 5).

### 3.6. DuoX Decreases NO Production in LPS-Induced RAW 264.7 Cells

**Figure 6:**
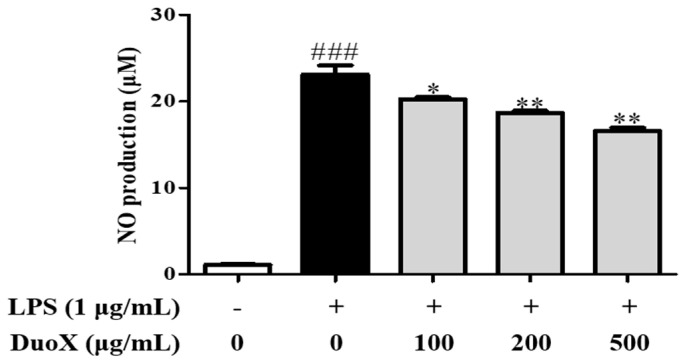
Effects of DuoX on nitric oxide (NO) production in lipopolysaccharide (LPS)-induced RAW 264.7 cells. RAW 264.7 cells were seeded in 24-well plates and pretreated with DuoX for 30 min, followed by stimulation with LPS for 24 h. Data are represented as the mean ± standard error of mean (n = 4). ### *p* < 0.001 compared with the LPS-untreated control; * *p* < 0.05, ** *p* < 0.01 compared with the LPS-treated group.

To assess the anti-inflammatory activity of DuoX, NO production was measured in LPS-stimulated RAW 264.7 macrophages. LPS treatment significantly increased NO levels in macrophages compared to those in the control group. However, pretreatment with DuoX markedly reduced NO production compared to that in the DEX-induced group, indicating that DuoX may suppress macrophage-mediated inflammatory responses (Figure 6).

### 3.7. DuoX Decreases COX-2 Protein Expression and MAPK Pathway Phosphorylation in LPS-Induced RAW 264.7 Cells

**Figure 7:**
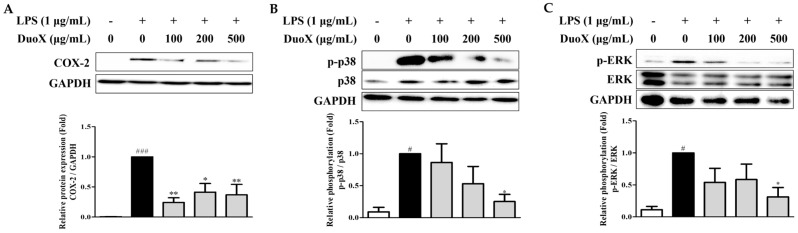
Effects of DuoX on cyclooxygenase-2 (COX-2) protein expression and mitogen-activated protein kinase (MAPK) pathway phosphorylation in lipopolysaccharide (LPS)-induced RAW 264.7 cells. RAW 264.7 cells were pretreated with DuoX for 30 min, followed by stimulation with LPS for 24 h. Total cell lysates were collected and analyzed to assess COX-2 protein expression (**A**) and the phosphorylation levels of p38 (**B**) and ERK (**C**) using western blotting, with GAPDH, total p38, and total ERK as loading controls. Data for p-p38 (n = 4) and COX-2 and p-ERK (n = 3) are presented as mean ± standard error of mean. # *p* < 0.05, ### *p* < 0.001 compared with the LPS-untreated control; * *p* < 0.05, ** *p* < 0.01 compared with the LPS-treated group.

To further investigate the anti-inflammatory mechanisms of DuoX, COX-2 expression and MAPK phosphorylation in LPS-stimulated RAW 264.7 cells were examined. LPS significantly increased COX-2 protein expression and p38 and ERK phosphorylation in these cells compared to those in the untreated group. However, pretreatment with DuoX effectively suppressed LPS-induced upregulation of COX-2 protein expression and p38 and ERK phosphorylation. Overall, these results suggest that DuoX exerts its anti-inflammatory effects by regulating COX-2 expression and inhibiting the upstream signaling pathway MAPK (p38 and ERK), thereby blocking key molecular pathways involved in inflammatory responses (Figure 7).

## 4. Discussion

This study focused on two major pathological processes associated with aging: sarcopenia and inflammation. During aging, muscle protein synthesis decreases while protein degradation increases, with the UPS–mediated by atrogin-1 serving as a key system of protein breakdown. Aging-associated inflammatory responses are primarily mediated by the MAPK and NF-κB signaling pathways [13,14]. Based on these molecular mechanisms, this study aimed to evaluate the muscle-protective and anti-inflammatory effects of DuoX.

DuoX is a novel dual-functional compound composed of *L. plantarum* beLP1, a postbiotic strain isolated from kimchi, and a plant-derived extract from the root of *C. intybus* L., which is rich in inulin. Previous studies have reported the anti-sarcopenic potential of beLP1, and inulin is a functional dietary fiber recognized by the U.S. FDA for its safety, and has been shown to exert antioxidant and anti-inflammatory effects in the gut [33].

In vitro, DuoX significantly inhibited the DEX-induced reduction in myotube diameter in C2C12 cells. Notably, morphological alterations induced by DEX were markedly restored in the high-dose DuoX group, with myotube diameter recovering to levels equivalent to those in the control group (Figure 1). The muscle-protective effects of DuoX were also confirmed in vivo. In DuoX-treated rats, the DEX-induced reductions in individual muscle weights—including the GA, TA, SOL, and PLA—were significantly attenuated (Figure 3). Additionally, DuoX prevented the decline in grip strength caused by DEX (Figure 4), suggesting its potential to alleviate DEX-induced muscle dysfunction. At the molecular level, the DEX-induced upregulation of atrogin-1 was suppressed by DuoX pretreatment in both the in vitro C2C12 cell model and the in vivo rat model (Figure 2 and Figure 5), indicating that DuoX protects against muscle degradation by inhibiting the UPS pathway. Based on previous studies reporting that atrogin-1 expression sensitively reflects the degree of sarcopenia, this study selected atrogin-1 as a representative marker of anti-sarcopenic effects and focused on analyzing its expression changes [8,39].

The anti-inflammatory effects were primarily evaluated using an in vitro model with RAW 264.7 macrophages. In these cells, DuoX significantly inhibited LPS-induced NO production (Figure 6). Moreover, western blot analysis revealed that DuoX pretreatment suppressed the LPS-induced expression of COX-2, a key marker of inflammation (Figure 7A). Phosphorylation of the MAPK pathway components p38 and ERK were also inhibited by DuoX (Figure 7B,C), providing molecular evidence for its anti-inflammatory effects. However, verification of the anti-inflammatory effects of DuoX in in vivo sarcopenia models remains a subject for future research, and such evaluations are planned to be conducted in upcoming studies.

Notably, p38 inhibition reduces the production of key pro-inflammatory cytokines such as TNF-α, IL-1β, and IL-6, independently of the NF-κB signaling pathway and suppresses NF-κB p65 K310 acetylation, directly inhibiting transcriptional activity. ERK inhibition further enhances anti-inflammatory effects by downregulating COX-2 expression [13,40]. However, the current study did not directly assess the NF-κB signaling pathway, indicating a need for further investigation. Future studies should expand these findings by analyzing additional muscle atrophy-related factors, such as FOXO3a, MuRF1, and AKT, and evaluating in detail the involvement of the NF-κB pathway to better elucidate DuoX-induced mechanism of action.

**Figure 8:**
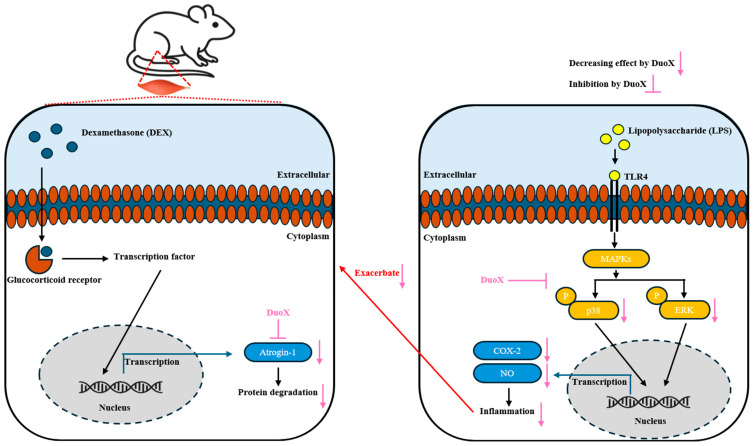
Schematic diagram of the anti-sarcopenic and anti-inflammatory effects of DuoX on dexamethasone (DEX)-induced sarcopenia in a rat model and lipopolysaccharide (LPS)-induced inflammation in RAW 264.7 cells.

This study employed DuoX, a complex compound primarily composed of beLP1 and inulin, both previously reported to exhibit anti-sarcopenic and anti-inflammatory effects. The results of our study confirmed that DuoX exerts both anti-sarcopenic and anti-inflammatory activities (Figure 8). However, as DuoX consists of more than ten components, it is challenging to delineate the individual contributions of each ingredient. Therefore, future studies should include comparative experiments with single components such as beLP1 or inulin alone to clarify their individual effects and possible synergistic interactions in the combined formulation.

Moreover, this study primarily focused on evaluating the anti-sarcopenic and anti-inflammatory effects of DuoX and did not include analyses of gut microbiota composition or metabolomics. Given the importance of postbiotics and inulin in modulating the intestinal microbial environment, future research should incorporate microbiological and metabolomic analyses to comprehensively elucidate DuoX-activated mechanisms [41,42,43,44]. Additionally, further evaluation of bioavailability, long-term safety, and metabolic fate is necessary for the clinical application of DuoX as a preventive functional food or therapeutic agent.

Even when considering these various limitations, this study experimentally confirmed that DuoX holds potential as a dual-functional compound capable of simultaneously modulating aging-related sarcopenia and inflammation, underscoring its clinical significance.

## 5. Conclusions

This study evaluated the dual preventive effects of DuoX, a complex functional compound combining the kimchi-derived postbiotic strain *L. plantarum* beLP1 and *C. intybus* L. root extract rich in inulin, on sarcopenia and inflammation. DuoX significantly alleviated DEX-induced muscle atrophy and functional decline in C2C12 myotubes and a rodent sarcopenia model, and it was confirmed to inhibit the activation of the UPS, a protein degradation pathway, by downregulating atrogin-1 expression. Additionally, in the RAW 264.7 macrophage model, DuoX exhibited anti-inflammatory effects by inhibiting NO production, COX-2 expression, and phosphorylation of MAPK signaling pathway elements (p38 and ERK) induced by LPS stimulation.

The study findings are meaningful in that they experimentally confirm DuoX as a complex functional compound capable of simultaneously regulating muscle atrophy and inflammation related to aging in both cellular and animal models. However, since DuoX is a mixture composed of multiple agents, it is necessary to elucidate the contribution of each component’s effects, and further evaluation of the molecular mechanisms related to inflammation is needed to gain a more detailed understanding of the anti-inflammatory pathways activated by DuoX. Therefore, the results of this study demonstrate that DuoX is a promising complex functional compound capable of concurrently alleviating aging-related muscle atrophy and inflammation. The study findings provide important foundational data for future clinical application research.

## Data Availability

The data that support the findings of this study are available from the corresponding author upon reasonable request.

## Abbreviations

COX-2	Cyclooxygenase-2
DEX	Dexamethasone
DMEM	Dulbecco’s modified Eagle’s medium
DW	Distilled water
ERK	Extracellular signal-regulated kinase
FBS	Fetal bovine serum
FDA	Food and Drug Administration
GA	Gastrocnemius
LPS	Lipopolysaccharide
MAPK	Mitogen-activated protein kinase
NF-κB	Nuclear factor kappa-light-chain-enhancer of activated B cells
NO	Nitric oxide
PBS	Phosphate-buffered saline
PLA	Plantaris
SOL	Soleus
TA	Tibialis anterior
UPS	Ubiquitin-proteasome system

## References

[1] Crimmins E.M. (2015). Lifespan and healthspan: Past, present, and promise. Gerontologist.

[2] Martinez R., Morsch P., Soliz P., Hommes C., Ordunez P., Vega E. (2021). Life expectancy, healthy life expectancy, and burden of disease in older people in the Americas, 1990–2019: A population-based study. Rev. Panam. Salud Pública.

[3] Najm A., Niculescu A.G., Grumezescu A.M., Beuran M. (2024). Emerging therapeutic strategies in sarcopenia: An updated review on pathogenesis and treatment advances. Int. J. Mol. Sci..

[4] Santilli V., Bernetti A., Mangone M., Paoloni M. (2014). Clinical definition of sarcopenia. Clin. Cases Miner. Bone Metab..

[5] Dodds R.M., Syddall H.E., Cooper R., Benzeval M., Deary I.J., Dennison E.M., Der G., Gale C.R., Inskip H.M., Jagger C. (2014). Grip strength across the life course: Normative data from twelve British studies. PLoS ONE.

[6] Mitchell W.K., Williams J., Atherton P., Larvin M., Lund J., Narici M. (2012). Sarcopenia, dynapenia, and the impact of advancing age on human skeletal muscle size and strength; a quantitative review. Front. Physiol..

[7] González-Blanco L., Bermúdez M., Bermejo-Millo J.C., Gutiérrez-Rodríguez J., Solano J.J., Antuña E., Menéndez-Valle I., Caballero B., Vega-Naredo I., Potes I. (2022). Cell interactome in sarcopenia during aging. J. Cachexia Sarcopenia Muscle.

[8] Clavel S., Coldefy A.S., Kurkdjian E., Salles J., Margaritis I., Derijard B. (2006). Atrophy-related ubiquitin ligases, atrogin-1 and MuRF1 are up-regulated in aged rat Tibialis Anterior muscle. Mech. Ageing Dev..

[9] Gumucio J.P., Mendias C.L. (2013). Atrogin-1, MuRF-1, and sarcopenia. Endocrine.

[10] Franceschi C., Garagnani P., Vitale G., Capri M., Salvioli S. (2017). Inflammaging and ‘Garb-aging’. Trends Endocrinol. Metab..

[11] Pugin J. (2012). How tissue injury alarms the immune system and causes a systemic inflammatory response syndrome. Ann. Intensive Care.

[12] Byars S.G., Voskarides K. (2020). Antagonistic pleiotropy in human disease. J. Mol. Evol..

[13] Moens U., Kostenko S., Sveinbjørnsson B. (2013). The role of mitogen-activated protein kinase-activated protein kinases (MAPKAPKs) in inflammation. Genes.

[14] Ci X., Li H., Yu Q., Zhang X., Yu L., Chen N., Song Y., Deng X. (2009). Avermectin exerts anti-inflammatory effect by down-regulating the nuclear transcription factor kappa-B and mitogen-activated protein kinase activation pathway. Fundam. Clin. Pharmacol..

[15] Binder E., Bermúdez-Silva F.J., André C., Elie M., Romero-Zerbo S.Y., Leste-Lasserre T., Belluomo l Duchampt A., Clark S., Aubert A. (2013). Leucine supplementation protects from insulin resistance by regulating adiposity levels. PLoS ONE.

[16] Schakman O., Dehoux M., Bouchuari S., Delaere S., Lause P., Decroly N., Shoelson S.E., Thissen J.-P. (2012). Role of IGF-I and the TNFα/NF-κB pathway in the induction of muscle atrogenes by acute inflammation. Am. J. Physiol.-Endocrinol. Metab..

[17] Coutinho A.E., Chapman K.E. (2011). The anti-inflammatory and immunosuppressive effects of glucocorticoids, recent developments and mechanistic insights. Mol. Cell. Endocrinol..

[18] Schakman O., Kalista S., Barbé C., Loumaye A., Thissen J.P. (2013). Glucocorticoid-induced skeletal muscle atrophy. Int. J. Biochem. Cell Biol..

[19] Wang B.Y.H., Hsiao A.W.T., Wong N., Chen Y.F., Lee C.W., Lee W.Y.W. (2023). Is dexamethasone-induced muscle atrophy an alternative model for naturally aged sarcopenia model?. J. Orthop. Transl..

[20] Tucureanu M.M., Rebleanu D., Constantinescu C.A., Deleanu M., Voicu G., Butoi E., Calin M., Manduteanu I. (2018). Lipopolysaccharide-induced inflammation in monocytes/macrophages is blocked by liposomal delivery of Gi-protein in-hibitor. Int. J. Nanomed..

[21] Kawasaki T., Kawai T. (2014). Toll-like receptor signaling pathways. Front. Immunol..

[22] Hankittichai P., Buacheen P., Pitchakarn P., Na Takuathung M., Wikan N., Smith D.R., Potikanond S., Nimlamool W. (2020). Artocarpus lakoocha extract inhibits LPS-induced inflammatory response in RAW 264.7 macrophage cells. Int. J. Mol. Sci..

[23] Yang G., Lee K., Lee M., Ham I., Choi H.-Y. (2012). Inhibition of lipopolysaccharide-induced nitric oxide and prosta-glandin E 2 production by chloroform fraction of Cudrania tricuspidata in RAW 264.7 macrophages. BMC Complement. Altern. Med..

[24] Maldonado R.F., Sá-Correia I., Valvano M.A. (2016). Lipopolysaccharide modification in Gram-negative bacteria during chronic infection. FEMS Microbiol. Rev..

[25] Park B.S., Lee J.O. (2013). Recognition of lipopolysaccharide pattern by TLR4 complexes. Exp. Mol. Med..

[26] Scott E., De Paepe K., Van de Wiele T. (2022). Postbiotics and their health modulatory biomolecules. Biomolecules.

[27] Proestos C. (2020). The benefits of plant extracts for human health. Foods.

[28] Vinderola G., Sanders M.E., Salminen S. (2022). The Concept of Postbiotics. Foods.

[29] Żółkiewicz J., Marzec A., Ruszczyński M., Feleszko W. (2020). Postbiotics—A Step Beyond Pre- and Probiotics. Nutrients.

[30] Jeong Y.-J., Kim J.-H., Jung Y.-J., Kwak M.-S., Sung M.-H., Imm J.-Y. (2024). KL-Biome (Postbiotic Formulation *of Lactiplantibacillus plantarum* KM2) Improves dexamethasone-induced muscle atrophy in mice. Int. J. Mol. Sci..

[31] Han S., Seo K.-H., Lee H.G., Kim H. (2023). Effect of Cucumis melo L. peel extract supplemented postbiotics on reprograming gut microbiota and sarcopenia in hindlimb-immobilized mice. Food Res. Int..

[32] Bueno E.B.T., Silva K.d.O., Mendes M.E.F., de Oliveira L.B., Menezes F.P.d., Imperador A.C., Correia L.F., Winkelstroter L.K. (2025). Postbiotics Derived from Lactic Acid Bacteria Fermentation: Therapeutic Potential in the Treatment of Muscular Complications in Inflammatory Bowel Disease. Fermentation.

[33] Xu Y., Liu X., Liu X., Chen D., Wang M., Jiang X., Xiong Z. (2021). The roles of the gut microbiota and chronic low-grade inflammation in older adults with frailty. Front. Cell. Infect. Microbiol..

[34] Li K., Zhang L., Xue J., Yang X., Dong X., Sha L., Lei H., Zhang X., Zhu L., Wang Z. (2019). Dietary inulin alleviates diverse stages of type 2 diabetes mellitus via anti-inflammation and modulating gut microbiota in db/db mice. Food Funct..

[35] Zou Y.-F., Li C.-Y., Fu Y.-P., Feng X., Peng X., Feng B., Li L.-X., Jia R.-Y., Huang C., Song X. (2022). Restorative effects of inulin from codonopsis pilosula on intestinal mucosal immunity, anti-inflammatory activity and gut microbiota of immunosuppressed mice. Front. Pharmacol..

[36] Guo L., Xiao P., Zhang X., Yang Y., Yang M., Wang T., Lu H., Tian H., Wang H., Liu J. (2021). Inulin ameliorates schizophrenia via modulation of the gut microbiota and anti-inflammation in mice. Food Funct..

[37] Choi J., Jeong E., Park H., Song H.-Y., Moon J., Kim M.-A., Koo B.S., Lee J.-H., Hong J.K., Han K.-I. (2025). Heat-Killed *Lactobacillus plantarum* beLP1 Attenuates Dexamethasone-Induced Sarcopenia in Rats by Increasing AKT Phosphorylation. Biomedicines.

[38] Farabegoli F., Santaclara F.J., Costas D., Alonso M., Abril A.G., Espiñeira M., Ortea I., Costas C. (2023). Exploring the anti-inflammatory effect of inulin by integrating transcriptomic and proteomic analyses in a murine macrophage cell model. Nutrients.

[39] Bodine S.C., Latres E., Baumhueter S., Lai V.K.-M., Nunez L., Clarke B.A., Poueymirou W.T., Panaro F.J., Na E., Dharmarajan K. (2001). Identification of ubiquitin ligases required for skeletal muscle atrophy. Science.

[40] Saha R.N., Jana M., Pahan K. (2007). MAPK p38 regulates transcriptional activity of NF-κB in primary human astrocytes via acetylation of p65. J. Immunol..

[41] Sawada D., Sugawara T., Ishida Y., Aihara K., Aoki Y., Takehara I., Takano K., Fujiwara S. (2016). Effect of continuous ingestion of a beverage prepared with Lactobacillus gasseri CP2305 inactivated by heat treatment on the regulation of intestinal function. Food Res. Int..

[42] Maehata H., Arai S., Iwabuchi N., Abe F. (2021). Immuno-modulation by heat-killed Lacticaseibacillus paracasei MCC1849 and its application to food products. Int. J. Immunopathol. Pharmacol..

[43] Birkeland E., Gharagozlian S., Birkeland K.I., Valeur J., Måge I., Rud I., Aas A.-M. (2020). Prebiotic effect of inulin-type fructans on faecal microbiota and short-chain fatty acids in type 2 diabetes: A randomised controlled trial. Eur. J. Nutr..

[44] Hiel S., Gianfrancesco M.A., Rodriguez J., Portheault D., Leyrolle Q., Bindels L.B., da Silveira Cauduro C.G., Mulders M.D., Zamariola G., Azzi A.-S. (2020). Link between gut microbiota and health outcomes in inulin-treated obese patients: Lessons from the Food4Gut multicenter randomized placebo-controlled trial. Clin. Nutr..

